# Chest computed tomography of a patient revealing severe hypoxia due to amniotic fluid embolism: a case report

**DOI:** 10.1186/1752-1947-4-55

**Published:** 2010-02-18

**Authors:** Hideaki Imanaka, Bunji Takahara, Harutaka Yamaguchi, Emiko Nakataki, Akiko Mano, Daisuke Inui, Jun Oto, Masaji Nishimura

**Affiliations:** 1 Department of Emergency and Critical Care, The University of Tokushima Graduate School, Kuramoto Tokushima, 770-8503, Japan

## Abstract

**Introduction:**

Amniotic fluid embolism is one of the most severe complications in the peripartum period. Because its onset is abrupt and fulminant, it is unlikely that there will be time to examine the condition using thoracic computed tomography (CT). We report a case of life-threatening amniotic fluid embolism, where chest CT in the acute phase was obtained.

**Case presentation:**

A 22-year-old Asian Japanese primiparous woman was suspected of having an amniotic fluid embolism. After a Cesarean section for cephalopelvic disproportion, her respiratory condition deteriorated. Her chest CT images were examined. CT findings revealed diffuse homogeneous ground-glass shadow in her bilateral peripheral lung fields. She was therefore transferred to our hospital. On admission to our hospital's intensive care unit, she was found to have severe hypoxemia, with SpO_2 _of 50% with a reservoir mask of 15 L/min oxygen. She was intubated with the support of noninvasive positive pressure ventilation. She was successfully extubated on the sixth day, and discharged from the hospital on the twentieth day.

**Conclusion:**

This is the first case report describing amniotic fluid embolism in which CT revealed an acute respiratory distress syndrome-like shadow.

## Introduction

Amniotic fluid embolism is one of the most severe complications in the peripartum period [1,2]. It is a rare but catastrophic illness with a high mortality rate. The onset is abrupt and fulminant, and there is no time to examine chest computed tomography (CT) images. However, in this case we had an opportunity to obtain a CT of a patient with suspected amniotic fluid embolism.

## Case presentation

**Figure 1:**
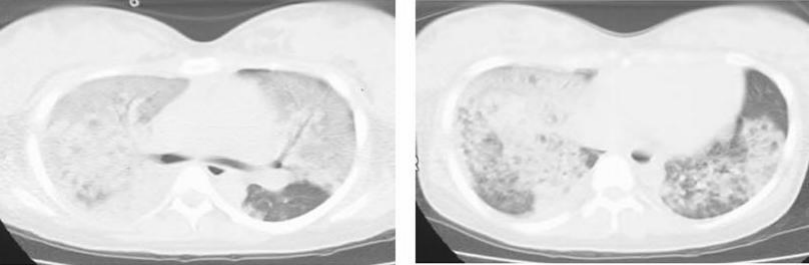
**The patient's chest CT prior to admission to the intensive care unit**. Homogeneous ground-glass opacity was observed in the peripheral field and spread out into the visceral pleura. Severe infiltration along the bronchoalveolar bundle was observed in the hilar portion.

A 22-year-old Asian Japanese primipara underwent a Cesarean section for cephalopelvic disproportion at 41 weeks and 2 days of her pregnancy. Although she showed hypertension, the course of her pregnancy had been otherwise uneventful. Her respiratory and hemodynamic conditions were stable during the operation. Three hours after the operation, she developed dyspnea, which was relieved by oxygen inhalation. The next morning, her respiratory condition deteriorated. Her dyspnea and cyanosis worsened rapidly and her SpO_2 _level decreased by 50% at room air. Although an attending physician first suspected air embolism or pulmonary thromboembolism, the results of her chest CT and ultrasonic cardiogram tests were not compatible with these disorders. Under a suspicion of amniotic fluid embolism, she was transferred to our hospital. The CT findings showed diffuse homogeneous ground-glass opacity in the bilateral peripheral fields and they were spread out up to the visceral pleura (Figure 1).

**Figure 2:**
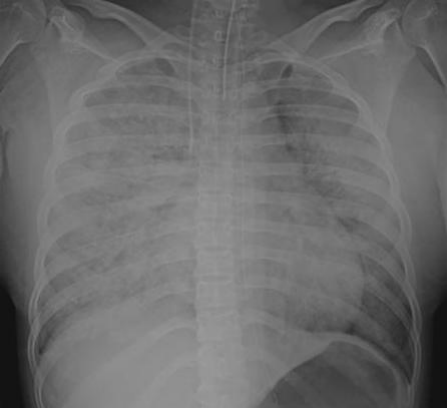
**The patient's chest X-ray on admission to the intensive care unit**. Diffuse infiltration was observed throughout the lungs.

On admission to our hospital's intensive care unit, her SpO_2 _level was 50% with a reservoir mask of 15 L/min oxygen and her respiratory rate was 54 breaths/min. Her consciousness was clear, her heart rate was 137 beats/min, her blood pressure was 109/83 mmHg, and her body temperature was 36.3°C. To make a tracheal intubation safe, we first applied noninvasive positive pressure ventilation at the following conditions: spontaneous and timed mode; F_I_O_2 _of 1.0; inspiratory positive airway pressure of 14 cmH_2_O; and expiratory positive airway pressure of 10 cmH_2_O. When her SpO_2 _increased to 90%, we performed intubation and found that there were no signs of laryngeal edema or aspiration. Mechanical ventilation was started in assist and control mode, F_I_O_2 _of 1.0, pressure control of 15 cmH_2_O, positive end expiratory pressure (PEEP) of 15 cmH_2_O, and tidal volume of 350 ml. Arterial blood gas analysis showed the following values: pH 7.06; PaO_2 _138 mmHg; PaCO_2 _69 mmHg; base excess -11.9 mmol/L; and lactate 3.0 mmol/L. Chest X-ray demonstrated diffuse infiltration throughout the lungs (Figure 2). There were no abnormal findings in her coagulation, kidney and liver functions. Cultures of tracheal aspirate, urine, and blood proved to be negative.

On the second day of admission, her brain natriuretic peptide (BNP) was high (2,194 pg/ml) and her left ventricular ejection fraction was 30% according to ultrasonic cardiogram. A 12-lead electrocardiogram showed sinus tachycardia with no signs of ischemia and infarction. On the sixth day, when her PaO_2 _improved to 112 mmHg at a setting of F_I_O_2 _0.25, pressure control 10 cmH_2_O and PEEP 4 cmH_2_O, her trachea was extubated. On the seventh day, her left ventricular ejection fraction recovered to 67%. She was discharged from the intensive care unit on the ninth day and from our hospital on the twentieth day after admission.

## Discussion

Amniotic fluid embolism is a rare but severe complication during pregnancy or shortly after delivery. Severe hypoxemia is usually an early symptom, and it is considered to be due to noncardiogenic pulmonary edema [3]. Left ventricular dysfunction develops coincidently. Our patient showed severe hypoxemia, and thoracic CTs were examined 18 hours after her delivery. We observed diffuse ground-glass shadows in her bilateral lung fields, which was suggestive of an early stage of acute respiratory distress syndrome. In addition, the enlarged pulmonary vasculature in the hilar regions of our patient may suggest the development of cardiogenic pulmonary edema. Just before the CT, echocardiography revealed 68% of ejection fraction. However, after her admission to the ICU, her BNP was 2,194 pg/ml and her ejection fraction decreased to 30%. We were not sure whether the CTs showed noncardiogenic or cardiogenic pulmonary edema, or both, because we did not follow the CT in series. The process was very quick and it was difficult to investigate the CT images.

To the best of our knowledge, there have been no reports of CT findings of this disease. Our patient's CTs absolutely showed diffuse ground-glass shadows. Although CT findings in this case were non-specific and were not useful tools for the diagnosis or treatment of amniotic fluid embolism, a follow-up of CT findings along our patient's recovery might have been interesting.

Amniotic fluid embolism is a clinical diagnosis primarily based on a constellation of clinical sequelae rather than on isolated signs and symptoms [3]. The current case was not typical amniotic fluid embolism in several points, including onset in a young primipara, delayed development of moderate respiratory symptoms followed by profound cardiorespiratory failure half day later, and a lack of signs of disseminated intravascular coagulation. It was therefore important to exclude other causes of sudden cardiorespiratory failure, including pulmonary thromboembolism, air embolism, anesthetic complications, aspiration of gastric contents, anaphylaxis, sepsis, myocardial infarction, and cardiomyopathy [3].

First, the CT and ultrasonic cardiogram findings did not correlate with pulmonary hypertension or right ventricular failure. Second, anesthetic complication or massive aspiration was unlikely because the intraoperative course was smooth and her consciousness was well maintained even when she had developed hypoxemia. Third, blood, sputum, urine and wound culture examination proved negative for infection. Finally, the absence of ischemic changes in 12-lead electrocardiogram and her rapid recovery of left ventricular function did not correlate with myocardial infarction or cardiomyopathy.

## Conclusion

In conclusion, when respiratory distress occurs in patients during the perinatal period, it is important to bear in mind the possibility of amniotic fluid embolism and to proceed with appropriate intensive care.

## Abbreviations

ARDS: acute respiratory distress syndrome; BNP: brain natriuretic peptide; CT: computed tomography; PEEP: positive end expiratory pressure.

## Consent

Written informed consent was obtained from the patient for the publication of this case report and any accompanying images. A copy of the written consent is available for review by the Editor-in-Chief of this journal.

## Competing interests

The authors declare that they have no competing interests.

## Authors' contributions

HI, BT and MN were responsible for the study conception and design. HY, DI and JO acquired the data and drafted the manuscript. AM and EN selected and commented on the imaging. All authors read and approved the final manuscript.
